# Algebraic topology-based machine learning using MRI predicts outcomes in primary sclerosing cholangitis

**DOI:** 10.1186/s41747-022-00312-x

**Published:** 2022-11-18

**Authors:** Yashbir Singh, William A. Jons, John E. Eaton, Mette Vesterhus, Tom Karlsen, Ida Bjoerk, Andreas Abildgaard, Kristin Kaasen Jorgensen, Trine Folseraas, Derek Little, Aliya F. Gulamhusein, Kosta Petrovic, Anne Negard, Gian Marco Conte, Joseph D. Sobek, Jaidip Jagtap, Sudhakar K. Venkatesh, Gregory J. Gores, Nicholas F. LaRusso, Konstantinos N. Lazaridis, Bradley J. Erickson

**Affiliations:** 1 Radiology, Mayo Clinic, Rochester, MN USA; 2 Biomedical Engineering and Physiology Graduate Program, Mayo Clinic Graduate School of Biomedical Sciences, Rochester, USA; 3 Division of Gastroenterology & Hepatology, Mayo Clinic, Rochester, MN USA; 4 Department of Medicine, Haraldsplass Deaconess Hospital, and Department of Clinical Science, University of Bergen, Bergen, Norway; 5 Norwegian PSC Research Center, Department of Transplantation Medicine, Division of Surgery, Inflammatory Medicine and Transplantation, Oslo University Hospital Rikshospitalet, Oslo, Norway; 6 Department of Radiology, Oslo University Hospital, Oslo, Norway; 7 Department of Gastroenterology, Akershus University Hospital, Nordbyhagen, Norway; 8 Toronto Centre for Liver Disease, University Health Network and Department of Medicine, University of Toronto, Toronto, Ontario Canada; 9 Department of Radiology, Haukeland University Hospital, Bergen, Norway; 10 Institute of Clinical Medicine, University of Oslo, Oslo, Norway; 11 Department of Diagnostic Imaging, Akershus University Hospital, Lørenskog, Norway

**Keywords:** Algorithm, Cholangitis (Sclerosing), Liver cirrhosis, Machine learning, Magnetic resonance imaging

## Abstract

**Background:**

Primary sclerosing cholangitis (PSC) is a chronic cholestatic liver disease that can lead to cirrhosis and hepatic decompensation. However, predicting future outcomes in patients with PSC is challenging. Our aim was to extract magnetic resonance imaging (MRI) features that predict the development of hepatic decompensation by applying algebraic topology-based machine learning (ML).

**Methods:**

We conducted a retrospective multicenter study among adults with large duct PSC who underwent MRI. A topological data analysis-inspired nonlinear framework was used to predict the risk of hepatic decompensation, which was motivated by algebraic topology theory-based ML. The topological representations (persistence images) were employed as input for classification to predict who developed early hepatic decompensation within one year after their baseline MRI.

**Results:**

We reviewed 590 patients; 298 were excluded due to poor image quality or inadequate liver coverage, leaving 292 potentially eligible subjects, of which 169 subjects were included in the study. We trained our model using contrast-enhanced delayed phase T1-weighted images on a single center derivation cohort consisting of 54 patients (hepatic decompensation, *n* = 21; no hepatic decompensation, *n* = 33) and a multicenter independent validation cohort of 115 individuals (hepatic decompensation, *n* = 31; no hepatic decompensation, *n* = 84). When our model was applied in the independent validation cohort, it remained predictive of early hepatic decompensation (area under the receiver operating characteristic curve = 0.84).

**Conclusions:**

Algebraic topology-based ML is a methodological approach that can predict outcomes in patients with PSC and has the potential for application in other chronic liver diseases.

**Supplementary Information:**

The online version contains supplementary material available at 10.1186/s41747-022-00312-x.

## Key points

- Algebraic topology-based machine learning can extract informative features.
- This approach can indicate visual patterns of the liver associated with hepatic decompensation in patients affected with primary sclerosing cholangitis (PSC).
- The novel workflow was validated on a multicenter cohort of PSC patients.

## Background

Primary sclerosing cholangitis (PSC) is a rare, slowly progressive, and heterogeneous disease with a varied phenotypic spectrum, consisting of chronic cholestatic liver condition characterized by inflammation and fibrosis of the extra and/or intrahepatic bile ducts, which lacks effective medical therapy. It is strongly associated with inflammatory bowel disease. Over time, it can lead to progressive hepatic fibrosis and complications stemming from portal hypertension (*i.e.*, hepatic decompensation) [1, 2]. Hence, predicting patient outcomes and the conduct of therapeutic clinical trials is essential. There is a limited portfolio of validated biomarkers that can be used in clinical practice to identify PSC patients at risk of adverse outcomes and for the conduct of clinical trials, either for patient stratification or as surrogate endpoints [2, 3].

Magnetic resonance imaging (MRI), particularly magnetic resonance cholangiopancreatography (MRCP) is routinely used to diagnose PSC and monitor for PSC-related complications [4]. Qualitative MRI/MRCP prognostic scoring systems, generated by individual radiologists, are hampered by suboptimal performance, limited reproducibility, and poor generalizability [5–8]. Elastography is a quantitative MRI technology that can predict adverse outcomes in those with PSC. However, this technology is not widely available [9, 10]. The severity of intrahepatic bile duct dilation, quantified using MRCP images, correlates with markers of the Mayo PSC risk score. However, quantitative MRCP metrics has not been widely studied or demonstrated to predict outcomes. Using laboratory data, a machine learning (ML) approach was able to predict adverse outcomes in those with PSC and performed better than other traditional predictive tools such as the Mayo PSC risk score [11]. However, it remains to be seen if quantitative ML from imaging could further enhance our ability to predict clinically relevant events [12].

A potential quantitative technique that may prove advantageous for understanding PSC is topological data analysis. TDA is a modern method for evaluating large-scale data that employs methodologies from geometry and algebraic topology [6, 7]. Complex relationships within multidimensional data can be retained and jointly considered by examining geometric and topological aspects of the data originating from various distance metrics placed on the data. Some important concepts and methods in algebraic topology include the notion of modeling data as a metric space (a set of points along with a measure of how apart any two points are), the definitions of what a simplex and simplicial complex are, the notion of filtrations, and the method of persistent homology [13–15] (Supplementary file 1). TDA is already being used by researchers in a variety of domains, including computational biology, to discover new knowledge from massive datasets [16, 17], and indeed, numerous studies have shown the effectiveness of topological data analysis (TDA) in a variety of medical applications [13–15, 18].

Given this promising technique and the unmet need to better predict adverse outcomes in those with PSC, we sought to determine if MRI features analyzed by TDA, and algebraic topology-based ML can predict adverse outcomes in those with PSC.

## Methods

### Inclusion/exclusion criteria

Five centers participated in this study: Mayo Clinic Rochester, three Norwegian centers (University Hospitals of Berge, Oslo, and Akershus), and the University of Toronto. The inclusion criteria for this study were a diagnosis of large duct PSC and availability of a contrast-enhanced T1-weighted MRI sequence of the abdomen obtained in the axial plane [19]. Specifically, contrast-enhanced T1-weighted MRI images in the delayed phase (after 3 or 5 minutes after the intravenous injection) were used. These T1-weighted images were obtained either as a three-dimensional liver acceleration volume acquisition (LAVA) sequence or a three-dimensional volumetric interpolated breath-hold examination (VIBE) sequence, depending on the MRI scanner (1.5 or 3 T) using conventional extracellular contrast medium or hepatobiliary contrast medium (Supplementary file 1). MRI exams were performed between 2007 and 2018. Exams for a patient were excluded if they did not include the entire liver within their field of view or if they exhibited discontinuous coverage of the liver due to the significant gaps between slices in their coverage of the liver.

### Clinical data

Clinical information was collected at the time of the MRI. Generally, patients were followed in the clinic every 6–12 months, and MRCP was performed annually. This included key laboratory tests including serum alkaline phosphatase expressed relative to that laboratory test’s upper limit of normal within 3 months of the baseline MRI. Hepatic decompensation was defined as the development of ascites, hepatic encephalopathy, or variceal hemorrhage [12]. The baseline clinical features for the derivation and validation cohort were compared using the “CreateTableOne” function of the tableone R package (https://www.rdocumentation.org/packages/tableone/versions/0.13.2). Categorical variables were summarized using counts and percentages and compared using *χ*^2^ testing. Continuous variables were expressed as medians and interquartile ranges and compared with Wilcoxon rank sum testing.

### Semiautomated liver segmentation

Liver segmentations were generated using a semiautomated approach, involving an initial segmentation by a deep learning model followed by corrections by a human (Y.S.), if needed. All the segmentation was done by an informatics fellow, but the project was supervised by B.E., a board-certified radiologist with 28 years of experience, who saw some of the segmentations. For each patient, one large patch was constructed from 25 small patches containing primarily liver pixels (*i.e.*, at least 80 percent of pixels falling within the liver) (Supplementary file 1).

### Feature extraction

Adams et al. proposed persistence images as a means to vectorize persistence images for ML applications in 2017 [20]. We chose their persistence image technique because of its ability to engage with a larger range of ML algorithms. We extracted interval values from the *persistent homology* filtration (birth time and death time) and constructed a *persistent image* to utilize this information in the ML task (Supplementary file 1). The features of the persistence image were extracted using the local binary pattern (LBP) feature extractor. These features were used to train a decision tree classifier to predict whether the patient developed hepatic decompensation within a year or not [21]. We used persistence diagrams for visual pattern representation.

**Fig. 1:**
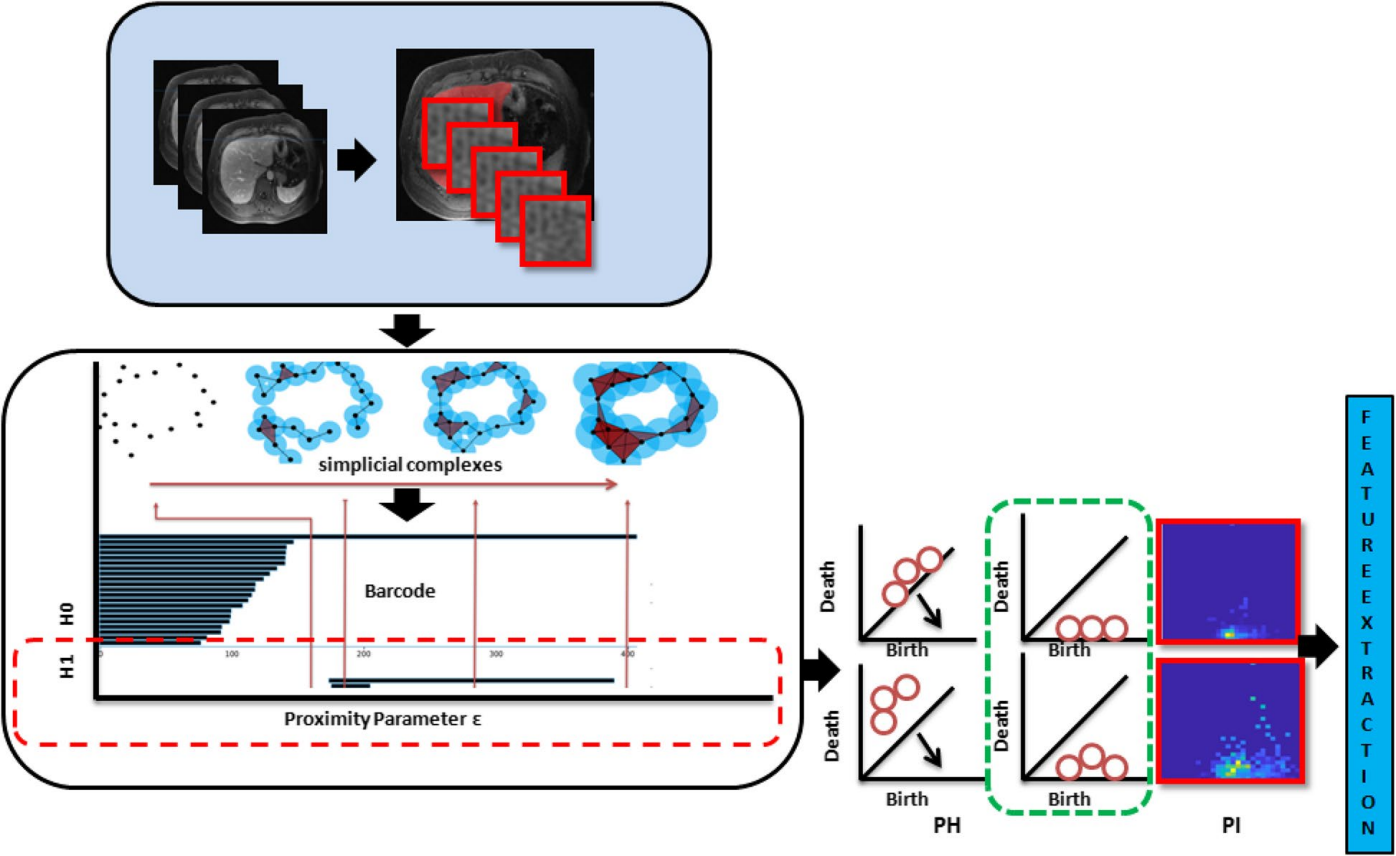
Workflow of algebraic topology-based machine learning with imaging signal as input

After applying the semiautomated approach, we segmented the whole liver and used an algorithm to create patches (blue box). We concatenate all the patches and then apply algebraic topology to generate the barcode (white box). We extracted all the interval values (birth and death values) with a diagonal identity line to create a persistence diagram and rotated diagram (green box) and then the persistent image. Using the persistent image, we extracted features for traditional supervised ML (Fig. 1).

### ML model

The extracted features from the persistence image served as input for our classifier. We used scikit-learn (version 0.24.2) to train a decision tree model (sklearn.tree.DecisionTreeClassifier) to discriminate MRIs from patients developing hepatic decompensation using a stratified *k*-fold cross-validation approach (*k* = 5). A patient was considered to have the outcome of interest if they were noted to have hepatic decompensation within 1 year from the baseline MRI. We used default parameters to train the model (criterion = gini; splitter = best; max_depth = none; min_samples_split = 2; min_samples_leaf = 1; min_weight_fraction_leaf = 1; max_features = none; random_state = 33; max_leaf_nodes = none; min_impurity_decrease = 0.0; min_impurity_split = 0; class_weight = none; ccp_alpha = none). The metrics to evaluate our model were balanced accuracy, weighted F1 score, area under the receiver operating characteristic curve (AUROC), and average precision score.

## Results

We reviewed 590 patients with PSC who underwent an MRI exam with the required sequences. Two hundred ninety-eight individuals were excluded due to poor image quality or inadequate coverage of the liver, leaving 169 subjects that were included in the study. Fifty-four patients from Mayo Clinic comprised the derivation group (hepatic decompensation, *n* = 21; no hepatic decompensation, *n* = 33). The validation cohort included 115 subjects (hepatic decompensation, *n* = 31; no hepatic decompensation, *n* = 84) from multiple centers (Mayo Clinic, *n* = 68; three Norwegian Centers, *n* = 41; Toronto, *n* = 6). The clinical features of the cohort are shown in Table 1.

**Table 1:** Baseline characteristics of patients

	Derivation cohort/training (*n* = 54)	Validation cohort (*n* = 115)	*p* value
Females	21 (38.9%)	39 (33.9%)	0.619
Inflammatory bowel disease presence	49 (90.7%)	94 (81.7%)	0.166
Age (years)	38.5 [24.0, 52.8]	49.7 [32.3, 58.9]	0.019
PSC duration (years)	3.26 [0.41, 11.28]	0.32 [0.00, 4.69]	0.002
SAP/ULN	2.50 [1.26, 4.24]	1.72 [1.03, 3.07]	0.039
Total bilirubin (mg/dL)	0.90 [0.50, 2.90]	0.80 [0.50, 1.86]	0.624
Platelets (× 10^9^/L)	267 [169, 326]	238 [167, 338]	0.800

Data are given as median [interquartile range] with the exception of the number and percentage of females and of patients with inflammatory bowel disease. For some patients, the MRI/MRCP which established their diagnosis of PSC was used in this study. The date of the MRI used in the study was time zero or the baseline. Therefore, the duration of PSC for some subjects was 0 years

*MRI* Magnetic resonance imaging, *MRCP* Magnetic resonance cholangiopancreatography, *PSC* Primary sclerosis cholangitis, *SAP/ULN* Serum alkaline phosphatase relative to the upper limit of normal within three months of the baseline magnetic resonance imaging

The “derivation” group was used for the cross-validation analyses. The “validation cohort” served as the test data set.

### Hepatic decompensation patterning

In topological data analysis, persistent homology is described via the persistence barcodes described above. There is a distinct barcode for each homology persistence vector space from which we are able to track the Betti numbers of the complexes for every value of *ε* [20].

**Fig. 2:**
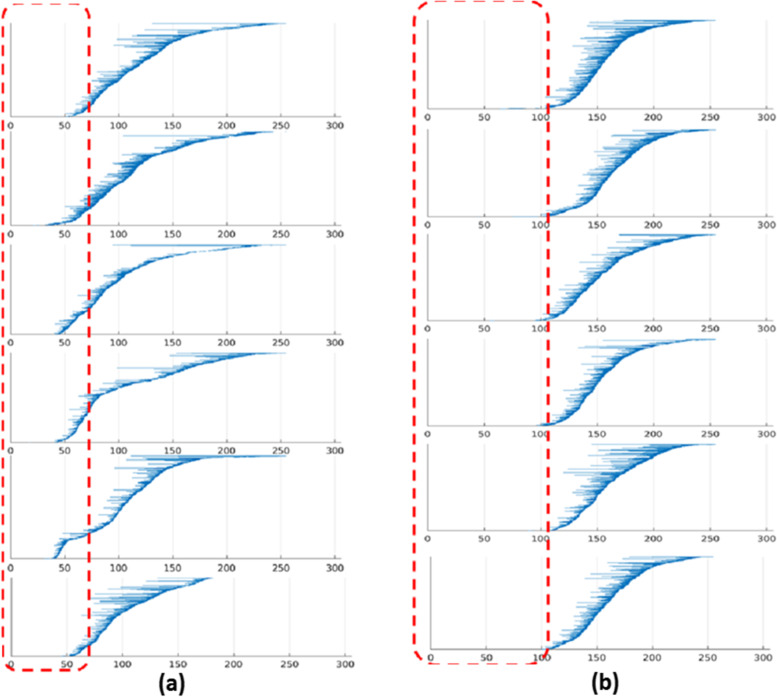
A barcode representation of hepatic decompensation status within a year. The horizontal axis line corresponds to the parameter *ɛ*, and the vertical axis line is the ordering of the homology generators. Note that the vertical placement is only introduced for display and does not have any intrinsic meaning. **a** Patients with hepatic decompensation within a year group exhibit Betti numbers typically between 0 and 50. **b** Patients without hepatic decompensation exhibit Betti numbers between 0 and 100. With the red box-based indicator, we can see a clear difference between the 1-dimensional homologies durations in both categories

The range of Betti numbers is relatively small (from 0 to 50) in the patients developing hepatic decomposition within a year (Fig. 2a), whereas the range of Betti numbers is quite large (0–100) in those not developing hepatic decomposition (Fig. 2b). As a result, we focus on the differences between the patients developing hepatic decompensation within 1 year and those not developing hepatic decompensation within 1 year.

**Fig. 3:**
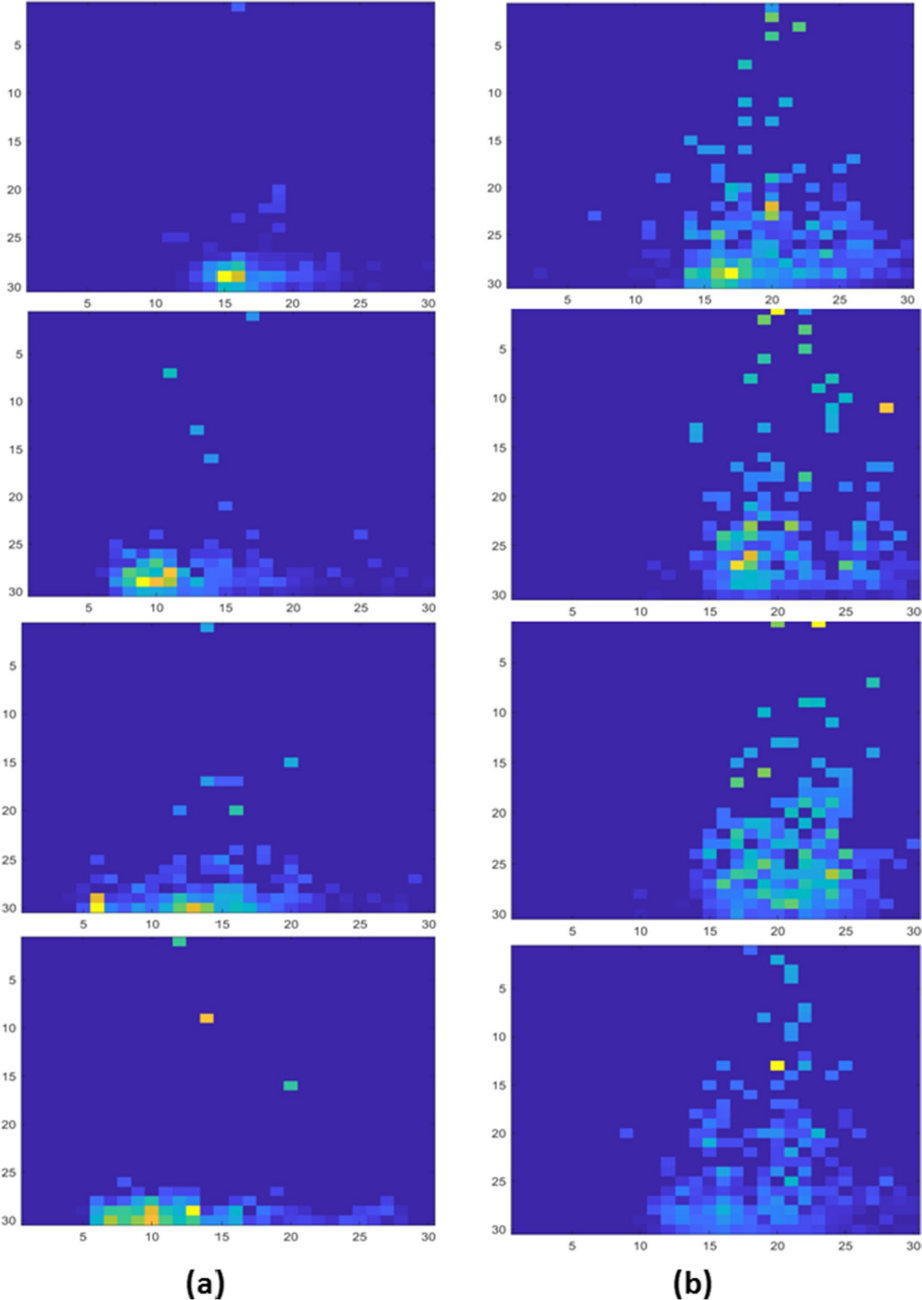
A persistence image representation of hepatic decompensation within a year. Persistence image is showing topologic data analysis stable vectorization for both (**a**) patients developing hepatic decompensation within a year and (**b**) patients not developing hepatic decompensation within a year

Betti numbers display a difference in geometrical pattern between the two groups. General trends can be observed through visual inspection. Patients with hepatic decompensation within a year patients have higher intensity values at lower persistence pixels and lower intensity values at higher persistence pixels. This shows that these patients have a clustered pattern (Fig. 3a). Those without hepatic decompensation within a year have a substantially larger variation in their persistence pixel intensities, indicating greater variability in these patients (Fig. 3b).

### ML classifier

**Fig. 4:**
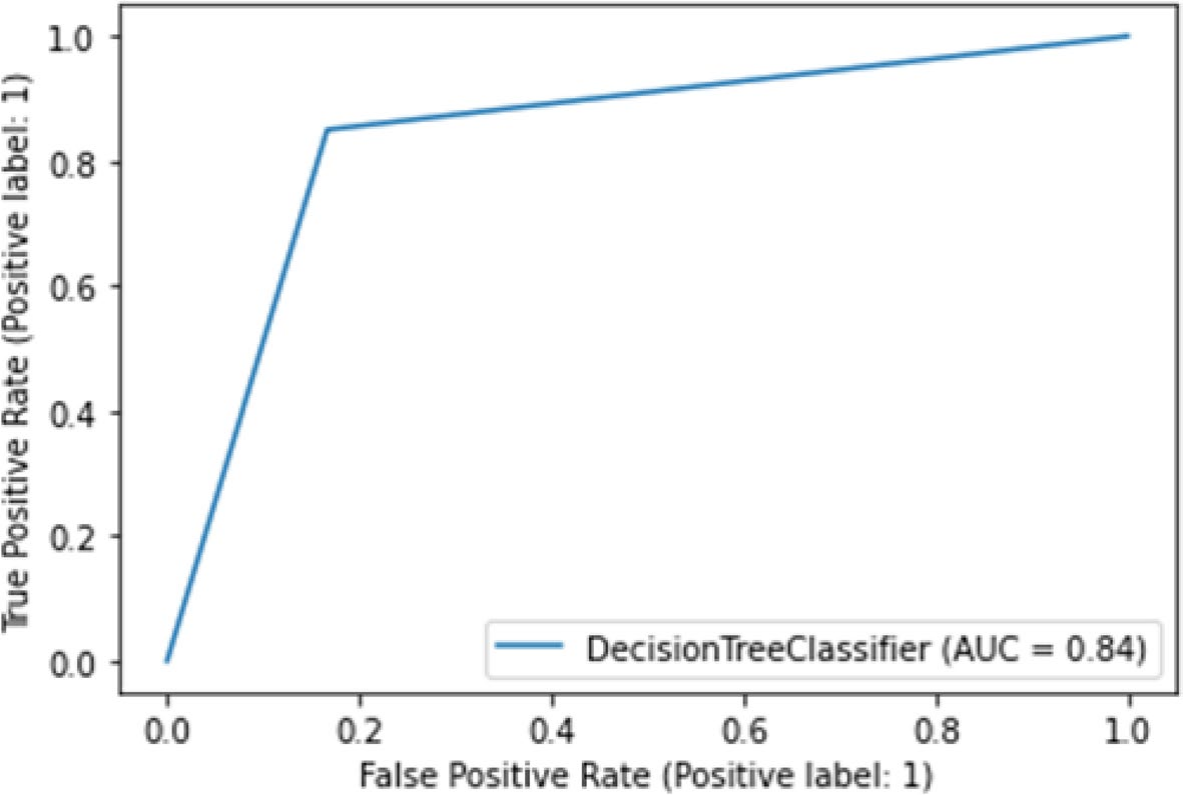
Receiver operating characteristic curve for validation data

Table 2 reports the results of the cross-validation analysis in the derivation cohort. The decision tree model can discriminate between the two classes with a median (± median absolute deviation) of 0.80 (± 0.12) balanced accuracy, 0.70 (± 0.08) F1 score, 0.74 (± 0.04) average precision, and 0.80 (± 0.012) AUROC. We applied this algorithm to a separate multicenter cohort and obtained an AUROC of 0.84 (Fig. 4).

**Table 2:** Median (± median absolute deviation) values for metrics obtained during a 5-fold stratified cross-validation evaluation of the decision tree classifier in the derivation cohort

Metric	Fold 1	Fold 2	Fold 3	Fold 4	Fold 5	Median (± median absolute deviation)
Balanced accuracy	0.8	0.91	0.67	0.67	0.8	0.80 (± 0.12)
F1 score	0.8	0.90	0.69	0.69	0.77	0.70 (± 0.08)
Average precision	0.74	0.93	0.69	0.69	0.81	0.74 (± 0.04)
AUROC	0.8	0.91	0.67	0.67	0.80	0.80 (± 0.12)

*AUROC* Area under the receiver operating

## Discussion

We developed a multicenter, proof-of-concept study that illustrates the merits of algebraic topology-based ML in generating predictive models from MRI exams. This study demonstrates this approach can analyze a vast amount of imaging data, detect distinct imaging patterns in those with advanced disease, and accurately predict short-term outcomes in patients with PSC.

We observed unique imaging patterns in those patients who did and did not develop early hepatic decompensation. For example, the ranges of Betti numbers in those who developed hepatic decompensation patients were very small (from 0 to 50) compared to those without hepatic decompensation (from 0 to 100). Small Betti numbers represent very low persistence, whereas large Betti numbers indicate more persistent topological features. It is possible these pattern differences represent morphologic changes associated with advancing fibrosis and portal hypertension [22–24].

Surrogate markers that can predict disease severity and outcomes for patients with PSC are needed [2]. ML and quantitative MRI data are promising approaches to predict outcomes in these patients. Laboratory data analyzed with ML has been shown to predict hepatic decompensation and survival after liver transplant for patients with PSC [12, 25]. A fully automated deep learning algorithm was shown to be able to analyze MRCP images and accurately detect patients with PSC compared to normal controls [26]. However, combining imaging and ML to predict outcomes in patients with PSC has not been conducted to date.

In this study, the algebraic topology-based ML approach used MRI data to create a model that predicted early hepatic decompensation. This algorithm continued to perform well when it was applied to a separate multicenter, international cohort (AUROC 0.84). Clinical applications for TDA and disease detection are emerging [16, 27–29]. To our knowledge, this is the first study to apply an ML algorithm based on algebraic topology and MRI data to predict the outcomes in patients with liver disease. This methodological approach may have the potential for the detection of other PSC-related complications such as cholangiocarcinoma and applications in other chronic liver diseases that are more common than PSC such as non-alcoholic fatty liver disease.

This study has several limitations. First, while our algorithm was validated in a multicenter cohort, it will be important to apply this model in larger studies given the heterogeneous disease spectrum associated with PSC and determine if this algorithm can perform well when there is incomplete image coverage of the liver or MRI exams that used series beyond what we required to train and validate the model. Second, as the first step in this proof-of-concept application of topological data analysis-based ML with imaging data, this model was designed to predict short-term outcomes. Future studies are needed to determine this model’s predictive performance for longer-term outcomes and if the incorporation of other clinical variables could enhance the model’s performance. Third, laboratory data was unavailable for many subjects and we were unable to compare the performance of our approach to other predictive markers such as the Mayo PSC risk score. Fourth, a relevant number of patients had to be excluded due to image quality, often due to the breath-hold nature of images leading to large discontinuities. Improved MRI methods may help to alleviate this challenge. Last, segmentation was semi-automated which requires expertise and can be time-consuming. Hence, future studies are needed to develop a fully automated approach to segmentation.

In summary, using topological data analysis-based ML, we discovered distinct patterns from MRI exams in those with PSC which enabled us to distinguish individuals who experienced early hepatic decompensation. The ability of this technology to create a persistent image that graphically characterizes the structural information derived from TDA has the potential for diverse diagnostic and prognostic clinical applications.

## Supplementary Information

**Additional file 1.**

## Data Availability

To protect patient privacy, individual-level patient data (including images) will not be shared. However, code supporting these analyses is available upon reasonable request.

## Abbreviations

AUROC: Area under the receiver operating characteristic curve
ML: Machine learning
MRCP: Magnetic resonance cholangiopancreatography
MRI: Magnetic resonance imaging
PSC: Primary sclerosing cholangitis
TDA: Topological data analysis
