# Supplementary material for: Algebraic topology-based machine learning using MRI predicts outcomes in primary sclerosing cholangitis
Source: Eur Radiol Exp. 2022 Nov 18;6:58. doi: 10.1186/s41747-022-00312-x (PMC9672219; doi:10.1186/s41747-022-00312-x)
Supplement: Supplementary file 1 — Additional file 1. [file 41747_2022_312_MOESM1_ESM.docx]

**MRI Acquisition Protocol**

The post contrast T1-weighted images were obtained with a three-dimensional T1 weighted spoiled gradient echo pulse sequence. The sequences were LAVA (Liver Acquisition with Volumetric Acquisition) on GE scanners and VIBE (Volume Interpolated Breathhold Examination)-DIXON on Siemens scanners 1.5T or 3T scanners. The parameters were TR (Repetition time)= 3 to 5 ms; TE (echo time)= minimum; TI (for GE scanners)= 7 to 24ms; FA (flip angle)= 11 to 15 degrees; NEX=1 ; slice thickness= 3 to 3.5mm (max of 4.5mm); slice gap= 0 to 2mm; FOV= 220 to 320mm.

The timing for the arterial phase was performed with bolus timing performed with 1mL gad injected at the rate of 1.5cc/sec followed by 20ml saline at 1.5cc/sec. After calculating the timing for arterial phase, the injection was performed at @ 1.5cc/sec of the determined volume for the patient (0.2 mL/kg  or 0.1 mmol/kg body weight) followed by 20ml saline @ 1.5cc/sec. The arterial phase and portal venous phases were acquired at 25s and 70s following the start of injection. Delayed phase was obtained at 3min or 5min post contrast injection. In the case of studies performed with Ge-EOB-DTPA (Eovist/Primovist), the hepatobiliary phase was obtained at 15-20 minute post contrast injection.

**Deep Learning Model Used for Liver Segmentation**

The model is a variation of the UNet architecture initially described in 2015 [1]. The model is a “2.5D” implementation, whereby the encoder input is an axial sliding window of 5 adjacent slices, followed by in succession, a full 3D bottom layer (unpadded convolutions in the z-dimension which compresses the 3D input to a 2D slice), a 2D decoder, and a fully connected classification layer to generate axial slice liver predictions. This type of model has demonstrated excellent performance on liver segmentation of computed tomography (CT) images [2, 3, 4] and MRI [5]. The model contained 3,731,809 trainable parameters and was trained on 228 manually segmented abdominal MRI scans with 80:20 training: validation split. Binary cross entropy was used as the loss function. Standard data augmentation was performed and included spatial shifts in the x and y dimensions, rotation, scaling, and addition of Gaussian noise. The model was trained until validation loss failed to improve for 10 consecutive epochs. The validation mean Dice Similarity Coefficient (DSC) of the model ultimately used in the semi-automated segmentation task was 0.87.

**Method to Generate a Patch from Image Segmentation**

The method for generating larger patches programmatically from smaller patches is described below.

Steps for Generating a Large Patch Composed of 25 Smaller Patches

i) Select a random slice number and random x and y coordinates

ii) Draw a 32x32 square on the image for that slice with corner with the random x and y coordinates chosen in step 1

iii) Check if at least 80% of the pixels within the 32x32 patch fall within the liver

If yes, add that patch to list of patches to be joined together, proceed to step 4

If no, go back to step 1 and try new random values for x and y and the slice number

iv) Repeat steps 1-3 until 25 small patches from the image have been found, each of which contains predominately liver pixels (>80% of patch pixels are from the liver)

v) Tile these 25 small patches to create a large patch which is 5 small patches x 5 small patches (160x160 pixels)

**Framework of TDA**

TDA is a modern method for evaluating large-scale data that employs methodologies from geometry and algebraic topology [6, 7]. Complex relationships within multi-dimensional data can be retained and jointly considered by examining geometric and topological aspects of the data originating from various distance metrics placed on the data. Topology encompasses a wide range of theories, and its impact on machine learning and deep learning is expanding all the time.

There are some concepts that have been reported by some previous research that includes three components.   First, a family of simplicial complexes indexed by a proximity parameter (there are two versions, the Cech and Vietoris-Rips complexes) can be constructed from collections of data points [8, 9]. The data is thus transformed into global topological objects. Second, the capability of viewing these complexes through the lens of algebraic topology, using persistent homology, an adaptation of the concept of homology to parameterized families. Third, it allows a data set's persistent homology to be expressed as a parameterized version of a Betti number known as a barcode. These are described in greater detail below.

i) Persistence Homology Filtration

Persistence homology (PH) is one of the most popular techniques for calculating topological properties of a space or images at various spatial resolutions. Key objects in topological data analysis are *filtered simplicial complexes*, sometimes just called *filtrations*. A filtered simplicial complex is an increasing family {X} (under inclusions of complexes) of simplicial complexes parametrized by elements of the non-negative real line.  *Homology* is a method for in an appropriate sense measuring the shape of a geometric object by counting holes or features of various dimensions.  Its output is a vector space for each non-negative integer *k* whose dimension (called the *k*-th Betti number) is the feature count for holes of dimension *k*.  This notion extends to filtered simplicial complex by producing a “persistence vector space”, the analogue of vector spaces, as well as the analogue of dimension, which is called a *persistence barcode* or *persistence diagram*. These Persistent Homology (PH) diagrams can be thought of as finite collections (unordered) of intervals, and one uses various algebraic combinations of the lengths of the intervals as well as their midpoints [7].

A barcode diagram depicts each persistence generator as a horizontal line that starts at the first filtration level and ends at the last filtration level, whereas a persistence diagram plots a point for each generator with its x-coordinate representing the birth time and its y-coordinate representing the death time. Visually, in two dimensions, each point is considered separately: we create a circle around those points with increasing radius until we locate certain intersections (birth time). One keeps creating larger circles until all of the points have been covered and all of the previously built constructions have been destroyed (death time). Finally, the birth time and death time are used, to produce the persistence diagram and barcode [8].

The creation of filtered chain complexes of vector spaces connected with actual point cloud datasets is one of the most essential functionalities of the JavaPlex library [http://appliedtopology.github.io/javaplex/] [9]. We employed sublevel set PH filtration in particular, the Vietoris–Rips filtration, which takes large patches as input and extracts topological features that indicate the data's key points [8, 9].

ii) Persistence Image (PI)

Metrics like Wasserstein or Bottleneck distance [12] can be used to compare the homology of persistence diagrams from different cases. Persistence landscapes [11], persistence barcodes [9], and persistent images (PIs) were introduced by Adams et al. in 2017 [13] to vectorize persistence diagrams for machine learning applications. Because of its capacity to interact with a wider range of machine learning algorithms and the flexibility to select only a few discriminatory pixels to retain in the limited space of a barcode, we chose their persistence image approach. A weighting function is used to assign a higher level of intensity to points that are more persistent. At each point, a Gaussian probability distribution with a chosen variance level is used. To form the PI with the chosen resolution, an N-by-N grid is superimposed over the surface. For machine learning and feature selection, the pixel intensities of the PI are used as a feature vector. For both viewing and storage, vectors from several component dimensions can be concatenated into a disease pattern barcode (Figure.1) [14].

**References**

1. Ronneberger, O., Fischer, P., & Brox, T. (2015, October). U-net: Convolutional networks for biomedical image segmentation. In International Conference on Medical image computing and computer-assisted intervention (pp. 234-241). Springer, Cham.

 2.  Li, X., Chen, H., Qi, X., Dou, Q., Fu, C. W., & Heng, P. A. (2018). H-DenseUNet: hybrid densely connected UNet for liver and tumor segmentation from CT volumes. IEEE transactions on medical imaging, 37(12), 2663-2674.

3. Weston, A. D., Korfiatis, P., Philbrick, K. A., Conte, G. M., Kostandy, P., Sakinis, T., ... & Erickson, B. J. (2020). Complete abdomen and pelvis segmentation using U‐net variant architecture. Medical physics, 47(11), 5609-5618.

4. Seo H, Huang C, Bassenne M, Xiao R, Xing L. (2019). Modified U-Net (mU-Net) with incorporation of object-dependent high level features for improved liver and liver-tumor segmentation in CT images. IEEE Trans Med Imag. 39(5), 1316-1325.

5.  Bobo MF, Bao S, Huo Y, Yao Y, Virostko J, Plassard AJ, Lyu I, Assad A., Abramson RG, Hilmes MA, Landman BA. (2018). Fully Convolutional Neural Networks Improve Abdominal Organ Segmentation. Proceedings of SPIE--the International Society for Optical Engineering, 10574, 105742V. https://doi.org/10.1117/12.2293751

6. Sauerwald, N., Shen, Y., & Kingsford, C. (2019). Topological data analysis reveals principles of chromosome structure throughout cellular differentiation. bioRxiv, 540716.

7. Carlsson, G., & Filippenko, B. (2020). Persistent homology of the sum metric. Journal of Pure and Applied Algebra, 224(5), 106244.

8.   Ghrist, R. (2008). Barcodes: the persistent topology of data. Bulletin of the American Mathematical Society, 45(1), 61-75.

9. Adams, H., & Tausz, A. (2011). Javaplex tutorial. Google Scholar.

10. Edelsbrunner, H., & Harer, J. (2008). Persistent homology-a survey. Contemporary mathematics, 453, 257-282.

11. Bubenik, P. (2015). Statistical topological data analysis using persistence landscapes. J. Mach. Learn. Res., 16(1), 77-102.

12. Amézquita, E. J., Quigley, M. Y., Ophelders, T., Munch, E., & Chitwood, D. H. (2020). The shape of things to come: Topological data analysis and biology, from molecules to organisms. Developmental Dynamics, 249(7), 816-833.

13. Adams, H., Emerson, T., Kirby, M., et al (2017). Persistence images: A stable vector representation of persistent homology. Journal of Machine Learning Research, 18.

14. Yan, Y., Ivanov, K., Mumini Omisore, O., Igbe, T., Liu, Q., Nie, Z., & Wang, L. (2020). Gait rhythm dynamics for neuro-degenerative disease classification via persistence landscape-based topological representation. Sensors, 20(7), 2006.


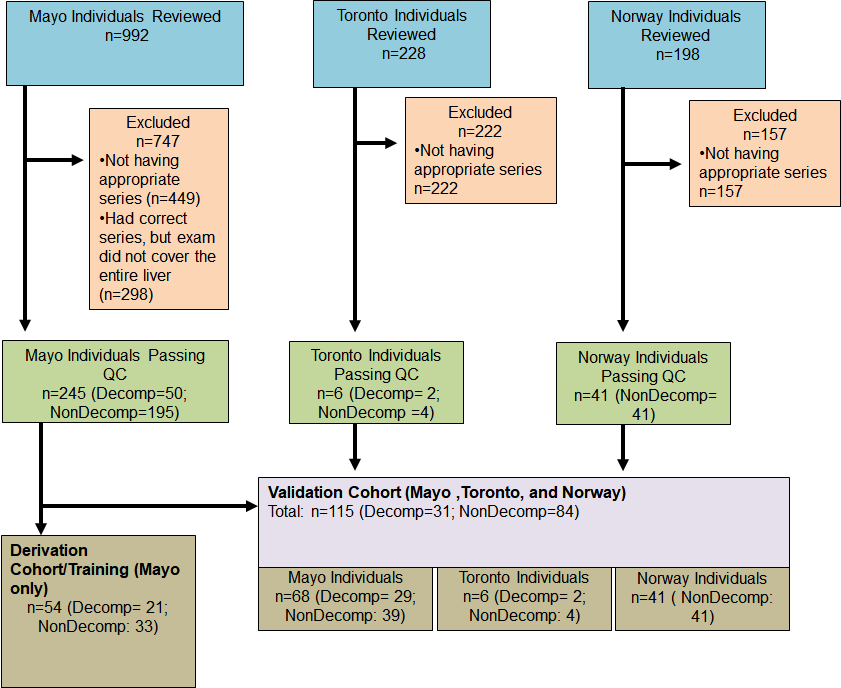


Supplementary figure 1. Flowchart describing patient selection for Derivation and Validation cohorts.
